# Cardiovascular Outcomes in a SELECT‐Like Obesity Cohort: Real‐World Insights From the Swedish AROS Database

**DOI:** 10.1111/dom.70899

**Published:** 2026-06-16

**Authors:** Viveca Ritsinger, Josefine Fagerström, Håkan Nero, Thomas Cars, Maria K. Svensson, Anna Norhammar

**Affiliations:** ^1^ Division of Cardiology, Department of Medicine Solna Karolinska Institute Stockholm Sweden; ^2^ Department of Research and Development Region Kronoberg Växjö Sweden; ^3^ Novo Nordisk Scandinavia AB Malmö Sweden; ^4^ Sence Research AB Uppsala Sweden; ^5^ Department of Medical Sciences, Renal Medicine Uppsala University Uppsala Sweden; ^6^ Uppsala Clinical Research Centre Uppsala Sweden; ^7^ Capio S:t Görans Hospital Stockholm Sweden

**Keywords:** antiobesity drug, cardiovascular disease, disease prevention, observational study, population study, real‐world evidence

## Abstract

**Background:**

In the SELECT trial, semaglutide reduced major adverse cardiovascular outcomes (MACE) in individuals with overweight or obesity and established cardiovascular disease (CVD) but without diabetes. However, real‐world cardiovascular event rates in comparable populations remain uncharacterised. We therefore aimed to (1) assess real‐world cardiovascular outcomes in a SELECT‐like cohort with obesity, (2) compare them to the general population and a broader population of individuals with obesity and (3) evaluate the cardiovascular preventive potential of semaglutide in this SELECT‐like cohort with obesity.

**Methods:**

We used healthcare registries and electronic health records from three Swedish regions (2013–2023), covering ~40% of the national population, to identify individuals aged ≥ 45 years with obesity (body mass index [BMI] ≥ 30 kg/m^2^) and established CVD but without diabetes, in alignment with the SELECT trial criteria. Cardiovascular outcomes in this *SELECT‐like obesity cohort* were compared with matched individuals from the general population as well as with a broader population of individuals with obesity, irrespective of CVD status or other comorbid conditions. To estimate the number needed to treat (NNT), the relative treatment effect of semaglutide observed in the SELECT trial was applied to the absolute risks identified in the *SELECT‐like obesity cohort*.

**Results:**

The *SELECT‐like obesity cohort* (*n* = 9652) had a mean (SD) age of 68.4 (11.3) years, a mean BMI of 33.1 kg/m^2^ (SD 3.5) and 57.9% were men. Most had a history of myocardial infarction (48.1%) or stroke (41.7%). Over a mean (SD) follow‐up of 5.4 (3.2) years, MACE occurred in 21.7%. Compared with the SELECT trial, this real‐world population had a higher mean age, had a higher proportion of females, and more often had a prior stroke. Applying the estimated effect of semaglutide from the SELECT trial, the NNT to prevent one MACE was 35 (95% CI, 24–66). Compared with the *General population cohort* (*n* = 48 260), the *SELECT‐like obesity cohort* had a higher burden of cardiovascular and obesity‐related comorbidities and an increased risk of adverse cardiovascular outcomes, including MACE (HR 2.3 [2.2–2.4]) and heart failure (HR 2.7 [2.5–2.9]).

**Conclusion:**

In a real‐world setting, despite the use of extensive preventive medications, individuals with obesity and CVD had a higher risk of adverse cardiovascular outcomes and mortality compared with the SELECT trial as well as the general population. These findings highlight the substantial disease burden and the need for improved secondary CVD prevention strategies.

## 1 Introduction

Nearly half of the world's population are overweight (body mass index [BMI] 25–29.9 kg/m^2^) or obese (BMI ≥ 30 kg/m^2^), contributing to 4 million deaths worldwide annually [1]. Obesity is associated with metabolic dysregulation and is a major risk factor for numerous conditions including cardiovascular disease (CVD) and cardiovascular mortality, independently of the risk mediated by obesity‐dependent risk factors [2]. Obesity‐related mortality is primarily driven by CVD (41%), followed by diabetes (10%), cancer (5%) and chronic kidney disease (5%) [1].

The global prevalence of obesity and obesity‐associated diseases continues to rise, highlighting the need for effective management strategies. Until recently, there were limited pharmacological treatments for obesity [3]. The Semaglutide Effects on Cardiovascular Outcomes in People With Overweight or Obesity (SELECT) trial showed that the glucagon‐like peptide‐1 receptor agonist semaglutide reduces the risk of major adverse cardiovascular events (MACE) in individuals with overweight or obesity, and established CVD but without diabetes [4]. Whilst this trial highlighted the relative benefits of the intervention, the absolute benefit will depend on characteristics and outcome rates in the target population in real‐world clinical care. Consequently, important knowledge gaps remain in our understanding of long‐term cardiovascular outcomes in the population that the SELECT trial aims to represent.

In this large, population‐based cohort study of a Swedish population, we address this gap by providing population‐level data on long‐term cardiovascular outcomes in individuals with obesity and prior CVD, but without diabetes. We compare real‐world risks of adverse cardiovascular outcomes and mortality with those reported in the SELECT trial. To further contextualise these findings, we also compare outcome rates with those of the general population and with a broader population of individuals with obesity, providing insights to inform public health strategies.

## 2 Methods

### 2.1 Data Source

Sweden's healthcare system is primarily publicly funded through general taxation and universally accessible, ensuring comprehensive coverage for all residents. In addition, each resident's healthcare interactions, including diagnoses, treatments and prescriptions, are recorded using a unique personal identification number (PIN), enabling extensive data collection across the population [5].

Data were obtained from multiple sources, including the National Patient Register (NPR), the Swedish Prescribed Drug Register, the National Cause of Death Register, Statistics Sweden (SCB) and regional healthcare data warehouses and electronic health records (EHRs) from Stockholm, Skåne and Dalarna regions [6, 7, 8]. Together, these sources cover approximately 40% of the Swedish population and provide a comprehensive dataset with detailed clinical and demographic information. Data were linked via each individual's PIN, pseudonymised and compiled into the AROS (Analysis of Real‐world data of patients with Obesity using Electronic Health Records and healthcare registries in Sweden) database. This integration of national and regional data provides a detailed and unique perspective on obesity‐related outcomes in a large, well‐defined Western European population.

The study was conducted following the Declaration of Helsinki and was approved by the Swedish Ethical Review Authority (approval numbers 2022‐00638‐01 and 2022‐03382‐02). Given the nature of the study, informed consent was not required.

### 2.2 Study Cohorts

#### 2.2.1 SELECT‐Like Obesity Cohort

The overall study cohort comprised all patients aged 45–93 years with a recorded BMI of ≥ 30 kg/m^2^ between 1 January 2013, and 30 June 2023. From this cohort, we identified the *SELECT‐like obesity cohort*, which included individuals with established CVD (history of myocardial infarction [MI], stroke or peripheral artery disease [PAD]) but without known diabetes (Figure S1A). In contrast to the SELECT trial, where PAD was defined based on symptoms or amputation, diagnosis and procedure codes were used for defining PAD in the *SELECT‐like obesity cohort*. Additional inclusion and exclusion criteria from the SELECT trial [5] were applied as closely as possible to ensure comparability (Table S1). Whilst the SELECT trial included individuals with a BMI ≥ 27 (from here on called the SELECT trial cohort), the AROS database does not contain data on patients with a BMI between 27 and 29.9 kg/m^2^ [9]. Inclusion criteria for the *SELECT‐like obesity cohort* required a stricter BMI threshold of ≥ 30 to specifically focus on individuals with obesity. Stratified analysis was carried out based on obesity classes: Class 1 obesity (30.0–34.9 kg/m^2^), class 2 obesity (35.0–39.9 kg/m^2^), class 3 obesity (≥ 40.0 kg/m^2^).

The index date in the SELECT trial was the date of randomisation. In the *SELECT‐like obesity cohort*, the index date was defined as the first date a BMI ≥ 30 kg/m^2^ was observed in the data. For patients who were hospitalised on that date, the index date was set as the date of discharge following fulfilment of the BMI criterion.

#### 2.2.2 General Population Cohort

To compare outcomes between the *SELECT‐like obesity cohort* and the general Swedish population, we created a matched cohort of population controls (hereafter referred to as *General population cohort*) from Statistics Sweden (SCB), consisting of a random selection of individuals residing in Stockholm, Skåne or Dalarna region. Patients in the *SELECT‐like obesity cohort* were matched 1:5 to controls by year of birth, sex, and region of residence, using replacement (i.e., one control could serve as a match for multiple cases). Each individual in the *General population cohort* was assigned the same index date as the corresponding case. By restricting matching to demographic variables only, the control group reflected the broader population, including both healthy individuals and those with comorbidities. This design enabled real‐world contextualisation of cardiovascular and mortality outcomes, highlighting differences between a high‐risk cohort with obesity and established CVD and the general population.

#### 2.2.3 General Obesity Cohort

To provide an additional clinically relevant comparator, we established a *General obesity cohort* comprising all individuals with a recorded BMI ≥ 30 kg/m^2^ in the AROS database, irrespective of CVD status or other comorbidities (Figure S1A). Patients in the *SELECT‐like obesity cohort* were matched 1:5 to controls from this broader obesity cohort on year of birth, sex, region of residence and year of first observed BMI ≥ 30 kg/m^2^, with replacement. Each matched control was assigned the same index date as the corresponding case. This approach allowed us to compare outcomes in the *SELECT‐like obesity cohort* with the wider population with obesity, providing a broader context for interpreting outcomes.

### 2.3 Patient Characteristics

Demographic information (age, sex, country of birth and region of residence) was obtained from SCB. Comorbid conditions were identified through diagnostic and procedure codes from the NPR, regional data warehouses, and EHRs. We included diagnoses and procedures recorded in specialised outpatient or inpatient care, in any diagnosis position, up to and including the index date. Information on medical treatments was retrieved from the Swedish Prescribed Drug Register, with medication use considered only if prescribed within the 365 days preceding or on the index date (Tables S2 and S3). Laboratory values and clinical measurements were extracted from EHRs, using the measurement closest to the index date within a 3‐year look‐back window.

### 2.4 Outcomes

Our outcomes mirrored the primary and secondary endpoints of the SELECT trial. A detailed description of all outcomes is provided in Table [Link], [Link]. All outcomes were assessed from the day after the index date onwards. Patients could experience the same type of event as their qualifying cardiovascular condition as a study outcome (e.g., a new myocardial infarction in a patient included based on prior myocardial infarction).

### 2.5 Statistical Analysis

Baseline characteristics were summarised across the groups using descriptive statistics, where continuous variables were reported as mean with standard deviation (SD) or median with interquartile range (IQR), and categorical variables were summarised as absolute numbers and percentages.

The cumulative incidence of the outcomes was estimated using the Aalen–Johansen method [10], which accounts for competing risks. Hazard ratios (HRs) were calculated using Cox proportional hazard regression, with the Fine‐Gray model employed to address competing risks [11]. The proportional hazards assumption was assessed using Schoenfeld residuals. To account for the clustered structure of the data (i.e., matching with replacement, where individuals could serve as controls for multiple cases), robust standard errors were used when calculating confidence intervals (CIs). Additional Cox models were used to compare obesity Class 2 and Class 3 with Class 1. Given differences in age and sex distributions across obesity groups, these analyses were adjusted for age and sex but not for other baseline covariates.

In our real‐world *SELECT‐like obesity cohort*, we observed a higher mean age and a different sex distribution compared with the SELECT trial cohort. To explore the potential impact of these differences, we conducted a secondary analysis using a weighting strategy. Post‐stratification weights, based on the product of age‐ and sex‐specific weights derived from the marginal age and sex distributions reported in the SELECT trial, were applied to adjust the age and sex distributions to that of the SELECT trial cohort, and all analyses were repeated in this reweighted cohort. This analysis was intended to provide additional insight into the extent to which demographic differences might influence the results.

Number Needed to Treat (NNT) for adverse cardiovascular outcomes was estimated by applying the relative effect from the SELECT trial [4] to the absolute risk observed in our *SELECT‐like obesity cohort* as follows:

$$\begin{array}{cc} \text{Absolute risk}\;\left(\mathrm{AR}\right)= & \text{cumulative incidence}\;\left(\text{AROS}\right)\\ & \times \text{hazard ratio}\;\left(\mathrm{HR}\right)\;\left(\text{from SELECT trial}\right) \end{array}$$

$$\text{Absolute risk reduction}\;\left(\mathrm{ARR}\right)=\text{cumulative incidence}\;\left(\text{AROS}\right)–\mathrm{AR}$$

NNT=1/ARR

NNT was calculated for all outcomes that reached statistical significance in the SELECT trial. To ensure comparability, we used the 48‐month event rate, consistent with the follow‐up period in the SELECT trial. Additionally, NNT was calculated separately for each BMI class. Because age and sex distributions differed between BMI classes, NNT calculations were weighted to match the distribution of the overall *SELECT‐like obesity cohort*, ensuring a more comparable analysis. We also report unadjusted NNT estimates. In calculating the NNT, we applied the overall HR from the SELECT trial across all BMI categories, rather than using separate HRs for each group. This approach was chosen because HRs for all outcomes and BMI groups were not available, and the effects of the primary endpoint in the SELECT trial appeared consistent between BMI groups, supporting the validity of using a single HR estimate [12]. Importantly, relative effect estimates such as hazard ratios are generally more transportable across cohorts than absolute measures, whereas absolute risk reductions and NNT are highly dependent on the underlying baseline risk [13]. Consequently, calculating NNT in a real‐world cohort using RCT‐derived relative effects and real‐world absolute risks is a useful approach that can provide more clinically relevant estimates [14, 15]. To assess the sensitivity of our results to missing HbA1c data, we conducted a complete case analysis restricted to patients with confirmed HbA1c < 48 mmol/mol and a multiple imputation analysis using predictive mean matching (Table S5). All statistical analyses were conducted using R version 4.3.3 (R Foundation for Statistical Computing, Vienna, Austria).

### 2.6 Role of the Funding Source

Novo Nordisk participated in study design, data interpretation and critical review of the manuscript. Novo Nordisk had no role in data collection and data analysis.

## 3 Results

A total of 284 412 patients aged 45–93 years with a recorded BMI ≥ 30 kg/m^2^ were identified. After applying the SELECT trial inclusion and exclusion criteria (Table S1), 9652 patients were classified as part of the *SELECT‐like obesity cohort*, representing 3.4% of the total obesity cohort (Figure S1). The mean BMI was 33.1 kg/m^2^ (SD 3.5) (Table 1), with 79.2% (*n* = 7645) classified as Class 1 obesity, 16.0% (*n* = 1546) as Class 2 obesity and 4.8% (*n* = 461) as Class 3 obesity. The most common qualifying cardiovascular event was myocardial infarction (MI) (48.1%), followed by stroke (41.7%) and peripheral artery disease (PAD) (6.0%). The mean time from the qualifying cardiovascular event to inclusion, defined as the first recorded BMI ≥ 30 kg/m^2^, was 7.3 years (SD 5.0) for MI, 7.3 years (SD 5.2) for stroke and 5.4 years (SD 5.5) for PAD. Additionally, 4.2% of patients had two or more qualifying conditions (Table 1).

**TABLE 1:** Baseline characteristics of the overall cohort in the SELECT trial, *SELECT‐like obesity cohort* (total and by classes of obesity), *General population cohort* and the *General obesity cohort*.

		SELECT trial population	SELECT‐like obesity cohort				General obesity cohort	General population cohort
			SELECT‐like obesity cohort (all)	Class 1 obesity (BMI 30–34.9)	Class 2 obesity (BMI 35–39.9)	Class 3 obesity (BMI ≥ 40)		
*N*		17 605	9652	7645	1546	461	48 074	48 260
Cardiovascular inclusion criteria								
Myocardial infarction	*n* (%)	11 908 (67.6)	4647 (48.1)	3710 (48.5)	740 (47.9)	197 (42.7)	N/A^a^	N/A^a^
Stroke		3135 (17.8)	4022 (41.7)	3143 (41.1)	665 (43.0)	214 (46.4)	N/A^a^	N/A^a^
Peripheral artery disease		777 (4.4)^b^	582 (6.0)	471 (6.2)	86 (5.6)	25 (5.4)	N/A^a^	N/A^a^
≥ 2 cardiovascular inclusion criteria		1433 (8.1)	401 (4.2)	321 (4.2)	55 (3.6)	25 (5.4)	N/A^a^	N/A^a^
Demographics								
Age (years)	Mean (SD)	61.6 (8.9)	68.4 (11.3)	68.9 (11.2)	67.0 (11.5)	65.1 (11.1)	68.3 (11.2)	68.4 (11.3)
45 to < 55	*n* (%)	4150 (23.6)	1324 (13.7)	963 (12.6)	264 (17.1)	97 (21.0)	6619 (13.8)	6620 (13.7)
55 to < 65		6727 (38.2)	2324 (24.1)	1810 (23.7)	386 (25.0)	128 (27.8)	11 608 (24.1)	11 620 (24.1)
65 to < 75		5362 (30.5)	3183 (33.0)	2539 (33.2)	493 (31.9)	151 (32.8)	15 896 (33.1)	15 915 (33.0)
75 to < 85		1318 (7.5)	2082 (21.6)	1700 (22.2)	314 (20.3)	68 (14.8)	10 353 (21.5)	10 410 (21.6)
≥ 85		48 (0.3)	739 (7.7)	633 (8.3)	89 (5.8)	17 (3.7)	3598 (7.5)	3695 (7.7)
Female		4872 (27.7)	4059 (42.1)	3058 (40.0)	748 (48.4)	253 (54.9)	20 213 (42.0)	20 295 (42.1)
Male		12 733 (72.3)	5593 (57.9)	4587 (60.0)	798 (51.6)	208 (45.1)	27 861 (58.0)	27 965 (57.9)
Region of birth								
North America	*n* (%)	4401 (25.0)	27 (0.3)	24 (0.3)	≤ 5	≤ 5	160 (0.3)	200 (0.4)
South America		1152 (6.5)	104 (1.1)	79 (1.0)	20 (1.3)	≤ 5	614 (1.3)	546 (1.1)
Europe		6507 (37.0)	8980 (93.0)	7110 (93.0)	1432 (92.6)	438 (95.0)	44 011 (91.6)	44 854 (92.9)
Africa		845 (4.8)	87 (0.9)	70 (0.9)	13 (0.8)	≤ 5	603 (1.3)	549 (1.1)
Asia		2201 (12.5)	431 (4.5)	343 (4.5)	76 (4.9)	12 (2.6)	2571 (5.3)	2015 (4.2)
Other		2499 (14.2)	23 (0.2)	19 (0.2)	≤ 5	≤ 5	112 (0.2)	94 (0.2)
Missing values		N/A	0	0	0	0	≤ 5	≤ 5
Body measurements								
BMI (kg/m^2^)	Mean (SD)	33.3 (5.0)	33.1 (3.5)	31.7 (1.3)	36.9 (1.4)	44.0 (4.0)	33.5 (3.8)	N/A^c^
27 to < 30	*n* (%)	5024 (28.5)	N/A^d^	N/A^d^	N/A^d^	N/A^d^	N/A^d^	N/A^c^
30 to < 35		7475 (42.5)	7645 (79.2)	100.0%	N/A	N/A	36 374 (75.7)	N/A^c^
35 to < 40		3346 (19.0)	1546 (16.0)	N/A	100.0%	N/A	8592 (17.9)	N/A^c^
≥ 40		1760 (10.0)	461 (4.8)	N/A	N/A	100.0%	3108 (6.5)	N/A^c^
Body weight (kg)	Mean (SD)	96.7 (17.7)	96.1 (14.8)	92.6 (11.6)	105.4 (14.0)	123.5 (20.8)	97.2 (16.0)	N/A^c^
Missing values^e^	*n* (%)	N/A	15 (0.2)	11 (0.1)	≤ 5	≤ 5	134 (0.3)	N/A^c^
Previous diseases and procedures^f^								
Coronary artery disease	*n* (%)	14 453 (82.1)	5608 (58.1)	4491 (58.7)	871 (56.3)	246 (53.4)	8491 (17.7)	5301 (11.0)
Myocardial infarction		13 439 (76.3)	5051 (52.3)	4035 (52.8)	794 (51.4)	222 (48.2)	3806 (7.9)	2335 (4.8)
Coronary revascularisation		11 849 (67.3)	4038 (41.8)	3281 (42.9)	605 (39.1)	152 (33.0)	4511 (9.4)	2775 (5.8)
Heart failure		4274 (24.3)^g^	1901 (19.7)	1463 (19.1)	336 (21.7)	102 (22.1)	5068 (10.5)	2354 (4.9)
Stroke		4108 (23.3)	4441 (46.0)	3484 (45.6)	719 (46.5)	238 (51.6)	3129 (6.5)	2378 (4.9)
Transient ischaemic attack		761 (4.3)	641 (6.6)	527 (6.9)	90 (5.8)	24 (5.2)	1837 (3.8)	1614 (3.3)
Hypertension		14 388 (81.7)	6699 (69.4)	5271 (68.9)	1089 (70.4)	339 (73.5)	22 481 (46.8)	13 565 (28.1)
Peripheral artery disease		1522 (8.6)^h^	856 (8.9)	700 (9.2)	120 (7.8)	36 (7.8)	1130 (2.4)	762 (1.6)
Atrial fibrillation		NR	2067 (21.4)	1636 (21.4)	329 (21.3)	102 (22.1)	6985 (14.5)	4335 (9.0)
MASLD^i^		1451 (8.2)	42 (0.4)	32 (0.4)	≤ 5	≤ 5	224 (0.5)	99 (0.2)
Diabetes mellitus		N/A^j^	N/A^j^	N/A^j^	N/A^j^	N/A^j^	10 780 (22.4)	4755 (9.9)
Bariatric surgery (incl. gastric bypass)^k^		NR	119 (1.2)	78 (1.0)	30 (1.9)	11 (2.4)	597 (1.2)	180 (0.4)
Glycaemic variables								
HbA1c (mmol/mol)	Mean (SD)	39.7 (3.7)	39.0 (4.0)	38.9 (4.0)	39.4 (4.0)	39.2 (4.0)	47.8 (14.5)	N/A^c^
< 39 (mmol/mol)	*n* (%)	NR	1724 (44.0)	1433 (44.5)	225 (42.1)	66 (41.0)	5210 (26.4)	N/A^c^
< 42 (mmol/mol)		NR	2826 (72.2)	2361 (73.3)	355 (66.5)	110 (68.3)	8473 (43.0)	N/A^c^
39–47 (mmol/mol)^l^		NR	2192 (56.0)	1788 (55.5)	309 (57.9)	95 (59.0)	5731 (46.3)	N/A^c^
42–47 (mmol/mol)^m^		NR	1090 (27.8)	860 (26.7)	179 (33.5)	51 (31.7)	2906 (23.5)	N/A^c^
HbA1c (%)	Mean (SD)	5.8 (0.3)	5.7 (0.4)	5.7 (0.4)	5.8 (0.4)	5.7 (0.4)	6.5 (1.3)	N/A^c^
< 5.7%	*n* (%)	5904 (33.5)	1724 (44.0)	1433 (44.5)	225 (42.1)	66 (41.0)	5210 (26.4)	N/A^c^
≥ 5.7%		11 696 (66.4)	2192 (56.0)	1788 (55.5)	309 (57.9)	95 (59.0)	14 489 (73.6)	N/A^c^
Missing values		N/A	5736 (59.4)	4424 (57.9)	1012 (65.5)	300 (65.1)	28 375 (59.0)	N/A^c^
Kidney function								
eGFR (mL/min/1.73 m^2^)	Mean (SD)	82.5 (17.4)	67.7 (18.1)	67.3 (17.8)	68.7 (19.0)	71.2 (19.5)	68.3 (18.6)	N/A^c^
Missing values	*n* (%)	N/A	692 (7.2)	490 (6.4)	159 (10.3)	43 (9.3)	10 351 (21.5)	N/A^c^
Lipid levels								
Total cholesterol (mmol/L)	Median (IQR)	4.0 (3.4–4.7)	4.2 (3.6–5.0)	4.2 (3.6–5.0)	4.4 (3.7–5.2)	4.4 (3.7–5.1)	4.8 (4.0–5.6)	N/A^c^
Missing values	*n* (%)	N/A	3534 (36.6)	2657 (34.8)	669 (43.3)	208 (45.1)	24 378 (50.7)	N/A^c^
LDL‐C (mmol/L)	Median (IQR)	2.0 (1.6–2.6)	2.4 (1.9–3.1)	2.4 (1.9–3.1)	2.5 (2.0–3.2)	2.5 (1.9–3.3)	2.9 (2.2–3.6)	N/A^c^
Missing values	*n* (%)	N/A	3862 (40.0)	2923 (38.2)	724 (46.8)	215 (46.6)	25 947 (54.0)	N/A^c^
HDL‐C (mmol/L)	Median (IQR)	1.1 (1–1.3)	1.2 (1.0–1.4)	1.2 (1.0–1.4)	1.2 (1.0–1.4)	1.2 (1.0–1.4)	1.2 (1.0–1.5)	N/A^c^
Missing values	*n* (%)	N/A	3858 (40.0)	2918 (38.2)	720 (46.6)	220 (47.7)	25 854 (53.8)	N/A^c^
Triglycerides (mmol/L)	Median (IQR)	1.5 (1.1–2.1)	1.4 (1.1–1.9)	1.4 (1.0–1.9)	1.5 (1.1–2.1)	1.5 (1.1–2.0)	1.5 (1.1–2.1)	N/A^c^
Missing values	*n* (%)	N/A	6015 (62.3)	4731 (61.9)	993 (64.2)	291 (63.1)	33 317 (69.3)	N/A^c^
Blood pressure lowering treatment^n^								
Any	*n* (%)	16 168 (91.8)	8409 (87.1)	6673 (87.3)	1331 (86.1)	405 (87.9)	33 405 (69.5)	22 122 (45.8)
Beta blockers		12 329 (70.0)	6314 (65.4)	5011 (65.5)	1007 (65.1)	296 (64.2)	19 392 (40.3)	11 804 (24.5)
ACE inhibitors		7915 (45)	4000 (41.4)	3154 (41.3)	662 (42.8)	184 (39.9)	13 661 (28.4)	8362 (17.3)
Angiotensin‐receptor blockers (ARB)		4706 (26.7)	2873 (29.8)	2279 (29.8)	446 (28.8)	148 (32.1)	13 907 (28.9)	8270 (17.1)
Calcium channel blockers (CCB)		5259 (29.9)	3037 (31.5)	2411 (31.5)	481 (31.1)	145 (31.5)	13 991 (29.1)	8628 (17.9)
Lipid lowering treatment^n^								
Any	*n* (%)	15 804 (89.8)	7095 (73.5)	5695 (74.5)	1088 (70.4)	312 (67.7)	18 710 (38.9)	12 049 (25.0)
Statins		15 369 (87.3)	6996 (72.5)	5617 (73.5)	1068 (69.1)	311 (67.5)	18 404 (38.3)	11 850 (24.6)
Ezetimibe		2314 (13.1)	576 (6.0)	489 (6.4)	70 (4.5)	17 (3.7)	893 (1.9)	551 (1.1)
Fibrates		477 (2.7)	48 (0.5)	38 (0.5)	7 (0.5)	≤ 5	270 (0.6)	117 (0.2)
PCSK‐9 inhibitors		335 (1.9)	20 (0.2)	20 (0.3)	≤ 5	≤ 5	11 (0.0)	17 (0.0)
Platelet aggregation inhibitors^n^								
Any	*n* (%)	15 130 (85.9)	6932 (71.8)	5560 (72.7)	1069 (69.1)	303 (65.7)	13 382 (27.8)	8935 (18.5)
Acetylsalicylic acid		13 691 (77.8)	6274 (65.0)	5039 (65.9)	962 (62.2)	273 (59.2)	12 532 (26.1)	8255 (17.1)
P2Y12 inhibitors		5912 (33.6)	1698 (17.6)	1412 (18.5)	224 (14.5)	62 (13.4)	2266 (4.7)	1261 (2.6)
Other platelet aggregation inhibitors^o^		179 (1.0)	287 (3.0)	228 (3.0)	42 (2.7)	17 (3.7)	330 (0.7)	240 (0.5)
Anticoagulants^n^								
Any	*n* (%)	2220 (12.6)	1913 (19.8)	1502 (19.6)	311 (20.1)	100 (21.7)	6913 (14.4)	3962 (8.2)
Vitamin K antagonists		670 (3.8)	1244 (12.9)	946 (12.4)	222 (14.4)	76 (16.5)	4280 (8.9)	2379 (4.9)
Direct oral anticoagulants		1510 (8.6)	727 (7.5)	607 (7.9)	93 (6.0)	27 (5.9)	2849 (5.9)	1690 (3.5)
Glucose‐lowering treatment^n^								
Any	*n* (%)	N/A^j^	N/A^j^	N/A^j^	N/A^j^	N/A^j^	12 009 (25.0)	5041 (10.4)
Insulin		N/A^j^	N/A^j^	N/A^j^	N/A^j^	N/A^j^	5274 (11.0)	1900 (3.9)
Non‐insulin (any)		N/A^j^	N/A^j^	N/A^j^	N/A^j^	N/A^j^	10 085 (21.0)	4204 (8.7)
Metformin		N/A^j^	N/A^j^	N/A^j^	N/A^j^	N/A^j^	8954 (18.6)	3754 (7.8)
Sulfonylureas		N/A^j^	N/A^j^	N/A^j^	N/A^j^	N/A^j^	1718 (3.6)	701 (1.5)
DPP‐4 inhibitors		N/A^j^	N/A^j^	N/A^j^	N/A^j^	N/A^j^	1202 (2.5)	559 (1.2)
GLP‐1 agonists		N/A^j^	N/A^j^	N/A^j^	N/A^j^	N/A^j^	895 (1.9)	318 (0.7)
SGLT2 inhibitors		N/A^j^	N/A^j^	N/A^j^	N/A^j^	N/A^j^	453 (0.9)	323 (0.7)
Other non‐insulin treatments		N/A^j^	N/A^j^	N/A^j^	N/A^j^	N/A^j^	566 (1.2)	221 (0.5)
Obesity‐management medications^n^, ^p^								
Orlistat	*n* (%)	NR	68 (0.7)	36 (0.5)	20 (1.3)	12 (2.6)	394 (0.8)	88 (0.2)
Bupropion/naltrexon		NR	≤ 5	≤ 5	≤ 5	≤ 5	9 (0.0)	≤ 5
Liraglutide^q^		NR	≤ 5	≤ 5	≤ 5	≤ 5	23 (0.0)	≤ 5

Abbreviations: ACE, angiotensin converting enzyme; BMI, body mass index; DPP‐4, dipeptidyl peptidase 4; eGFR, estimated glomerular filtration rate; GLP‐1, glucagon‐like peptide‐1 receptor agonists; HbA_1c_, glycated haemoglobin; HDL‐C, high‐density lipoprotein cholesterol; IQR, interquartile range; LDL‐C, low‐density lipoprotein cholesterol; MASLD, Metabolic Dysfunction‐Associated Steatotic Liver Disease; N/A, not applicable; NR, not reported in the SELECT trial; SD, standard deviation; SGLT2, sodium/glucose cotransporter 2.

^a^ Not an inclusion criterion for General population cohort and for the General obesity cohort. The proportion of patients in this group having these conditions is represented in this table under Previous disease and procedures.

^b^ Only symptomatic peripheral artery disease.

^c^ Not applicable as laboratory values and clinical measurements from electronic health records is not available for the General population cohort.

^d^ Not applicable as BMI values < 30 kg/m^2^ are not available in the AROS database.

^e^ Only BMI data is available, missing individual weight measurements.

^f^ At any time up to, and including, the index date.

^g^ Chronic heart failure.

^h^ Only symptomatic peripheral artery disease.

^i^ Possibly low coverage of this diagnosis in the Swedish clinical practice.

^j^ Not applicable as diabetes and/or use of glucose‐lowering medications was an exclusion criterion in the SELECT trial and SELECT‐like obesity cohort.

^k^ At any time prior to index.

^l^ HbA1c range classified as pre‐diabetes according to American Diabetes Association (ADA). Calculated among individuals without known diabetes mellitus.

^m^ HbA1c range for identification of individuals at high risk for diabetes according to the International Expert Committee (IEC). Calculated among individuals without known diabetes mellitus.

^n^ Within 12 months prior to, and including, the index date.

^o^ The category ‘Other’ includes dipyridamole (ATC: B01AC07).

^p^ GLP‐1 receptor agonists approved for obesity management had limited availability in Sweden during the study period. Reimbursement coverage was not available, and semaglutide 2.4 mg for obesity was not broadly accessible in Swedish pharmacies until May 2023.

^q^ The analysis included only Saxenda from the liraglutide ATC group (A10BJ02).

Within obesity classes, patients with Class 1 obesity had a higher mean age, included more males and had a larger proportion with coronary artery disease (CAD), MI and transient ischemic attack (TIA) compared with those in Classes 2 and 3. Hypertension and stroke were more often observed in Class 3 obesity, whilst a larger proportion of patients in Class 1 obesity used lipid‐lowering treatment and platelet aggregation inhibitors. Patients with Class 3 obesity had the highest use of anticoagulants and the highest prevalence of atrial fibrillation (Table 1).

**FIGURE 1:**
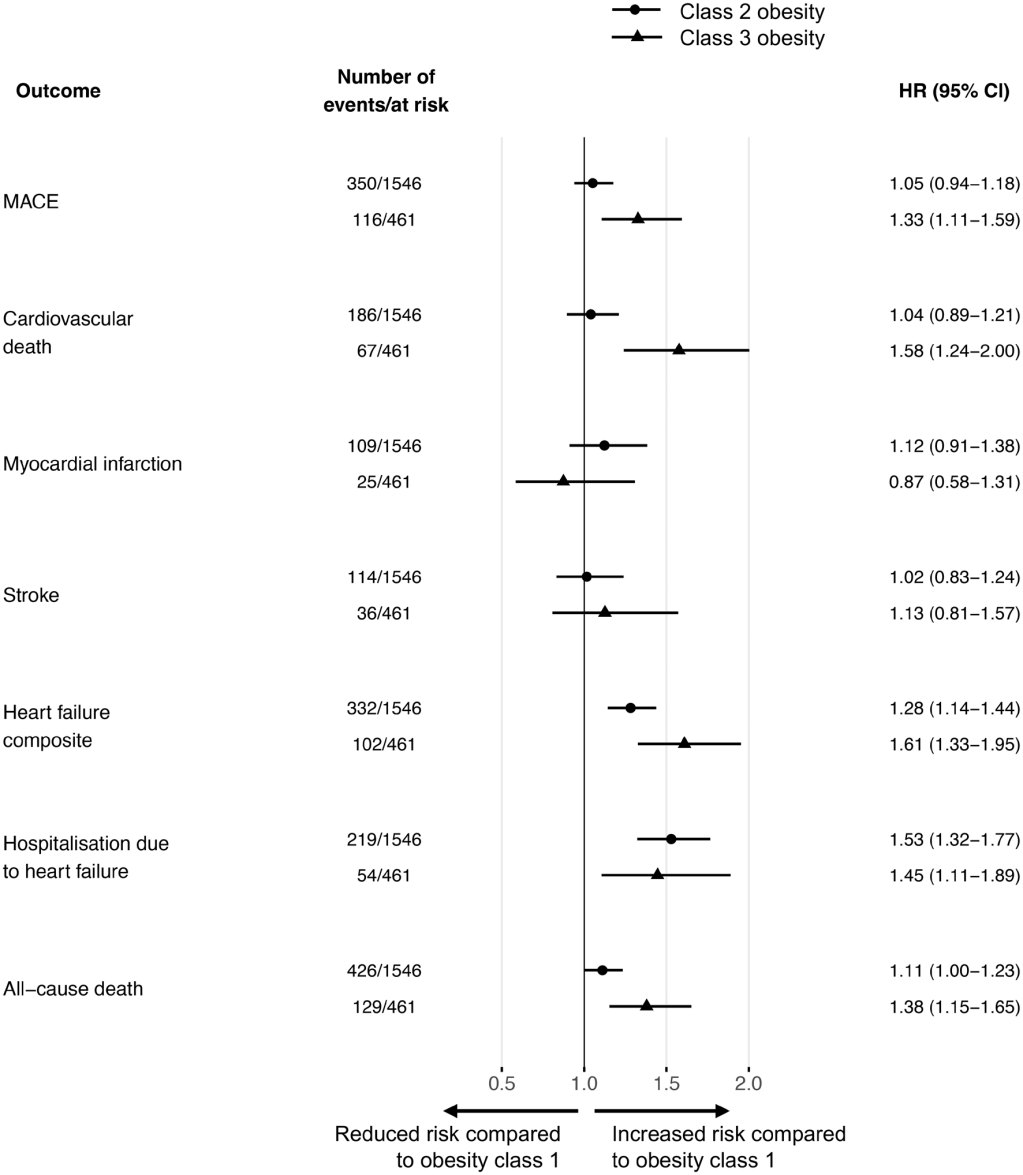
Rates of adverse cardiovascular outcomes in the SELECT‐like obesity cohort: obesity Classes 2 and 3 compared with Class 1 obesity. Major adverse cardiovascular events (MACE) were defined as a composite of cardiovascular death, myocardial infarction and stroke; heart failure composite was defined as a composite of hospitalisation due to heart failure or cardiovascular death. The definitions of all outcomes are provided in Methods and in Supporting Information. Note that this analysis is adjusted only for age and sex, making groups comparable with respect to these two variables only.

Over a mean follow‐up of 5.4 years (SD 3.2) for the *SELECT‐like obesity cohort*, MACE occurred in 21.7% (*n* = 2095), all‐cause death in 25.7% (*n* = 2483) and cardiovascular death in 12.1% (*n* = 1168). In age and sex adjusted analyses, patients with Class 3 obesity had a higher hazard of MACE (HR 1.33; 95% CI, 1.11–1.59), all‐cause death (HR 1.38; 95% CI, 1.15–1.65) and cardiovascular death (HR 1.58; 95% CI, 1.24–2.00) compared with those with Class 1 obesity (Figure 1). The highest hazard was observed for the heart failure composite outcome amongst patients with obesity Class 3 compared with Class 1 (HR 1.61; 95% CI, 1.33–1.95). No differences in MI or stroke hazards were observed across obesity classes (Figure 1).

Compared with the SELECT trial, the *SELECT‐like obesity cohort* had a lower proportion of males (57.9% vs. 72.3%) and higher mean age, with a mean age of 68.4 years (SD 11.3) compared with 61.6 years (SD 8.9) (Table 1). Whilst the proportion of patients with CAD and prior MI was higher in the SELECT trial, stroke was more common in the *SELECT‐like obesity cohort* (46.0% vs. 23.3%; Table 1).

**FIGURE 2:**
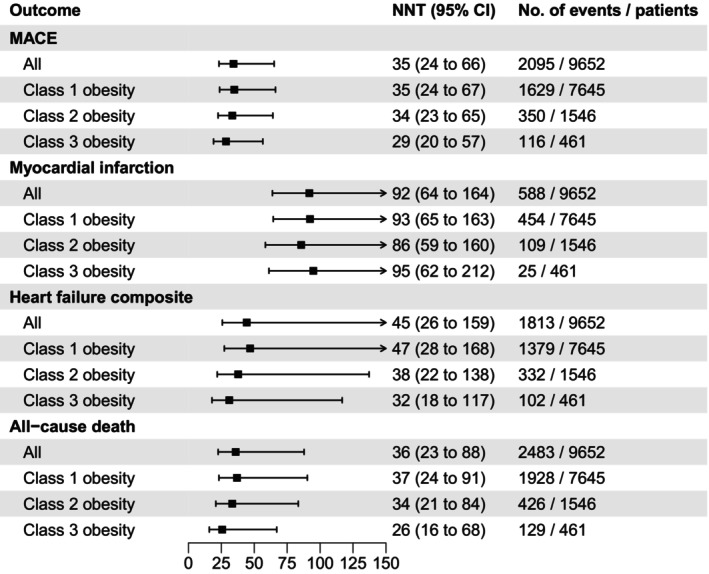
Number needed to treat (NNT) in the *SELECT‐like obesity cohort* grouped by BMI value when applying relative treatment effects from the SELECT trial (all groups are weighted to match the age and sex distribution of the overall *SELECT‐like obesity cohort*). Major adverse cardiovascular events (MACE) were defined as a composite of cardiovascular death, myocardial infarction and stroke; heart failure composite was defined as a composite of hospitalisation due to heart failure or cardiovascular death. The definitions of all outcomes are provided in Methods and in Supporting Information.

Applying the 48‐month relative treatment effect from the SELECT trial, we estimated the number needed to treat (NNT) in the *SELECT‐like obesity cohort* to prevent major outcomes. In the main analysis, outcomes were weighted to reflect the age and sex distribution of the overall *SELECT‐like obesity cohort*. Under this approach, the NNT was 35 (95% CI, 24–66) for MACE, 92 (95% CI, 64–164) for MI, 45 (95% CI, 26–159) for the heart failure composite and 36 (95% CI, 23–88) for all‐cause death (Figure 2). For comparison, both unweighted NNT estimates and values reweighted to match the age and sex distribution of the SELECT trial population are presented in Figures S2 and S3.

**FIGURE 3:**
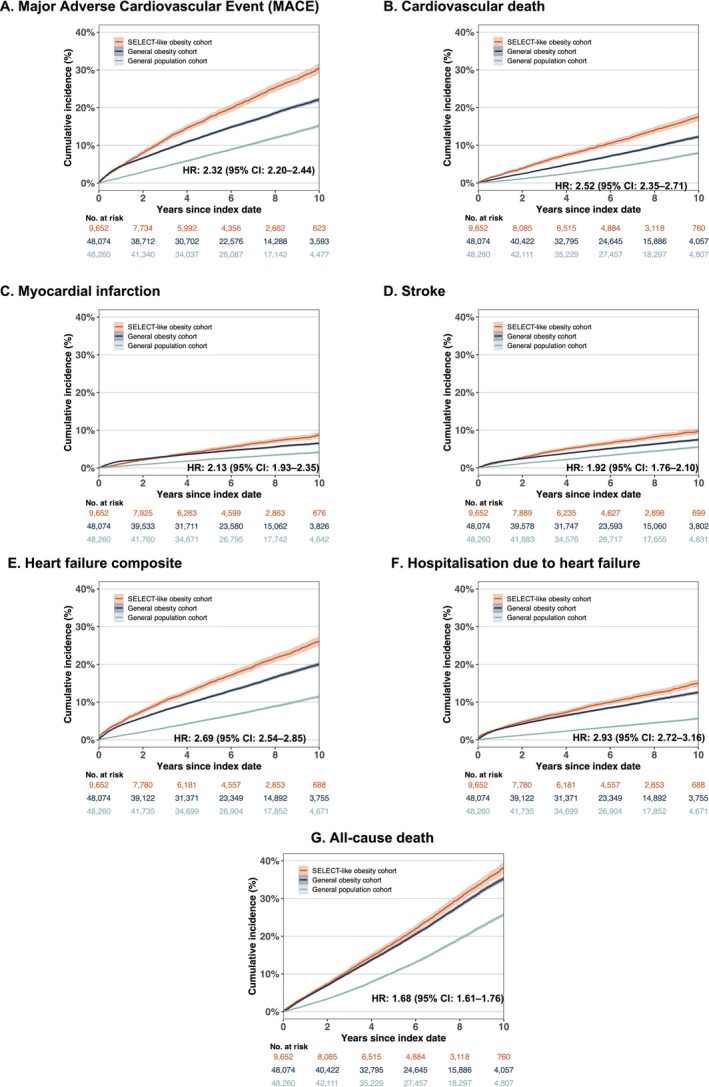
Time‐to‐first‐event analysis for adverse cardiovascular outcomes. Cumulative incidence and hazard ratios (competing risks taken into account) are shown for MACE (composite of cardiovascular death, myocardial infarction and stroke; competing risk: non‐cardiovascular death) (A), cardiovascular death (competing risk: non‐cardiovascular death) (B), myocardial infarction (competing risk: all‐cause death) (C), stroke (competing risk: all‐cause death) (D), heart failure composite (composite of hospitalisation due to heart failure or cardiovascular death; competing risk: all‐cause death) (E), hospitalisation due to heart failure (competing risk: all‐cause death) (F), and all‐cause death (no competing risks) (G). The definitions of all outcomes are provided in Methods and in Supporting Information. Hazard ratios (HRs) represent the comparison between the *SELECT‐like obesity cohort* and the *General population cohort*.

Patients in the *SELECT‐like obesity cohort* were matched based on age, sex and residence to individuals from the general population (*General population cohort* [*n* = 48 260; unique patients *n* = 46 089]), as well as to patients with a recorded BMI ≥ 30 kg/m^2^ without applying any additional selection criteria (*General obesity cohort* [*n* = 48 074; unique patients *n* = 42 976]) (Figure S1). The *SELECT‐like obesity cohort* had a substantially higher prevalence of all reported comorbidities (Table 1). The use of blood pressure‐lowering medications was highest in the *SELECT‐like obesity cohort* (87.1%), being nearly twice as high compared with the *General population cohort* (45.8%). Similarly, platelet aggregation inhibitor and lipid‐lowering treatment use were approximately three times higher (Table 1). During a mean follow‐up of 6.1 years (SD 3.1) for the *General population cohort*, MACE occurred in 10.3% (*n* = 4984), all‐cause death in 16.5% (*n* = 7981) and cardiovascular death in 5.1% (*n* = 2456). The analysed adverse cardiovascular events per 1000 patient‐years were consistently higher in the *SELECT‐like obesity cohort* compared with the *General population cohort* (Table 2). For MACE, the event rate was 40.4 (95% CI, 38.6–42.1) in the *SELECT‐like obesity cohort*, 17.0 (95% CI, 16.6–17.5) per 1000 patient‐years in the *General population cohort*, and 29.2 (95% CI, 28.5–29.9) in the *General obesity cohort* (Table 2). The largest differences between the *SELECT‐like obesity cohort* and the *General population cohort* were observed for hospitalisations due to heart failure (2.9‐fold higher in the *SELECT‐like obesity cohort*) and the heart failure composite outcome (2.7‐fold higher). Cumulative incidence of cardiovascular outcomes over 10 years amongst patients in the *General obesity cohort* was between that observed in the *SELECT‐like obesity cohort* and the *General population cohort* (Figure 3). Relative hazard estimates likewise showed the highest risks in the *SELECT‐like obesity cohort* compared with the *General population cohort*, whilst the *General obesity cohort* consistently displayed values between the two groups (Figure 3). In additional analyses adjusting for age and sex to match the SELECT trial, cumulative incidences for all outcomes were lower than in the unweighted analysis (Figure S4).

**TABLE 2:** Incidence rates for cardiovascular outcomes.

Outcome	Events per 1000 patient‐years (95% CI)		
	*SELECT‐like obesity cohort*	*General obesity cohort*	*General population cohort*
MACE	40.4 (38.6–42.1)	29.2 (28.5–29.9)	17.0 (16.6–17.5)
Cardiovascular death	20.9 (19.7–22.1)	14.1 (13.7–14.6)	8.1 (7.8–8.5)
Myocardial infarction	10.9 (10.1–11.8)	8.7 (8.3–9.0)	4.8 (4.5–5.0)
Stroke	12.7 (11.8–13.7)	9.7 (9.3–10.1)	6.2 (5.9–6.5)
Heart failure composite^a^	34.1 (32.5–35.7)	25.5 (24.9–26.1)	12.5 (12.1–12.9)
Hospitalisations due to heart failure	19.6 (18.4–20.8)	16.4 (15.9–16.9)	6.3 (6.0–6.6)
All‐cause death	44.4 (42.6–46.1)	40.8 (40.1–41.6)	26.4 (25.8–27.0)

Abbreviation: MACE, major adverse cardiovascular events.

^a^ Includes hospitalisations due to heart failure and cardiovascular death.

## 4 Discussion

In this large, real‐world Swedish healthcare data‐based analysis of patients with CVD and obesity there were several major findings. First, the risk of adverse cardiovascular outcomes and mortality was higher in real‐world settings compared with the SELECT trial. The estimated NNT to prevent one MACE in the *SELECT‐like obesity cohort* was 35, assuming the same relative effect as in SELECT, compared with 67 in the trial. Second, the combination of obesity and established CVD was associated with a substantially increased risk of cardiovascular events and mortality compared with both the general population and the broader population with obesity.

The SELECT trial demonstrated that the GLP‐1 receptor agonist semaglutide reduced major cardiovascular outcomes in patients with overweight or obesity (BMI ≥ 27 kg/m^2^) and established CVD [4]. Whilst randomised trials ensure strong internal validity, they may not be representative of patients seen in clinical practice. Contextualising trial cohorts against real‐world data is therefore critical. Consequently, the SCORE study applied SELECT‐like eligibility criteria to a large real‐world dataset to estimate the preventive potential of semaglutide on adverse clinical outcomes in US adults with established CVD and overweight/obesity but without diabetes, and observed a decreased risk of MACE and other obesity‐related complications [16]. In this study, applying SELECT inclusion and exclusion criteria to Swedish healthcare data identified a *SELECT‐like obesity cohort* of 9652 patients, who differed from the SELECT trial cohort by higher observed age and a larger proportion with a history of stroke. These differences likely reflect age‐related variation in disease presentation and suggest that patients with prior stroke may be underrepresented in cardiovascular outcome trials [17].

The real‐world *SELECT‐like obesity cohort* exhibited higher absolute risks of adverse outcomes than the SELECT trial cohort. After weighting for age and sex to align with the SELECT trial distribution, the excess risk was partly attenuated. Moreover, this study applied a BMI threshold of ≥ 30 kg/m^2^, the standard clinical definition of obesity, whereas the SELECT trial included patients with BMI ≥ 27 kg/m^2^. Notably, nearly one‐third of SELECT participants had a BMI of 27–30. Despite this lower BMI range, comorbidities such as CAD, MI, and heart failure were more prevalent in the SELECT trial cohort than in our real‐world cohort.

When applying the relative treatment effect from SELECT to our cohort, the estimated NNT for preventing one MACE was 35, substantially lower than in the trial. This estimation assumes that the relative treatment effect observed in SELECT is transportable to our cohort. Our cohort was older (mean age 68.4 vs. 61.6 years) and had a higher prevalence of prior stroke (46.0% vs. 23.3%) than the SELECT trial population. Whilst prespecified subgroup analyses in SELECT reported no significant treatment heterogeneity across age or BMI subgroups, the treatment effect appeared less pronounced in the subgroup with prior stroke alone [4]. Therefore, the NNT of 35 should be interpreted as a potential lower bound. This difference between the NNT in the trial and in the current study may also reflect the absence of patients with BMI of 27–30 in the current study. Nearly one‐third of SELECT participants fell within this overweight range, representing a lower‐risk subgroup. Although the SELECT trial population overall had a higher prevalence of established cardiovascular comorbidities, the higher BMI threshold in the current study likely selects for a population with greater obesity‐related risk, which may partly explain the higher observed event rates and consequently lower NNT. A Danish healthcare data‐based study emulating SELECT criteria reported similar findings, with lower NNT estimates than those observed in the RCT [4, 18]. Together, these results suggest that the preventive potential of semaglutide may be greater in clinical practice, where baseline risks are higher than in carefully selected trial cohorts. However, it should be acknowledged that not all of the excess cardiovascular risk observed in real‐world settings is necessarily modifiable through treatment, as it may partly reflect advanced disease burden less amenable to intervention.

Beyond the trial comparison, our analysis against the matched *General population cohort* demonstrated that the *SELECT‐like obesity cohort* is a clear high‐risk group. Event rates were more than doubled for most outcomes, with particularly pronounced excesses for heart failure hospitalisation and cardiovascular death. Despite widespread use of secondary preventive medications, residual risk remained considerable. To further contextualise our findings, we also examined a *General obesity cohort* including all individuals with obesity without applying any additional selection criteria. In line with prior literature documenting excess cardiovascular risk in obesity, this group had a significant burden of cardiovascular comorbidities, albeit lower than in the *SELECT‐like obesity cohort* [19, 20, 21, 22]. Characterisation of patients with obesity recorded in the real‐world clinical practice provides an important insight into the graded cardiovascular risk across patient groups. Amongst the nearly 300 000 individuals with obesity, only 9652 met the SELECT‐like criteria, underscoring that trial‐eligible patients represented a small, highly selected subset. As anticipated, outcomes in the *General obesity cohort* generally fell between those of the *SELECT‐like obesity cohort* and the general population, suggesting that obesity contributes to elevated cardiovascular risk, whilst the coexistence of obesity and established CVD identifies a particularly vulnerable subgroup. This reinforces the importance of viewing trial results not in isolation, but in the broader context of population‐level disease burden.

The strengths of this study include the near‐complete capture of healthcare interactions and outcomes through the linkage of national registries with regional EHRs. This comprehensive data integration provided a robust platform for assessing long‐term health outcomes in patients with obesity and established CVD but without diabetes. To enhance comparability, controls from both the general population and from a *General obesity cohort* were matched to cases. No additional selection criteria were applied, meaning the *General population cohort* and the *General obesity cohort* included individuals with and without obesity, diabetes, or prior CVD. This approach ensured that the control groups reflected the broader population distribution rather than a specifically matched healthy cohort, allowing for a more representative comparison of cardiovascular risk. An important strength of this study is the identification of SELECT‐like obesity patients and control groups; however, some limitations must be acknowledged. Whilst most SELECT trial selection criteria were met using this database, certain exclusion criteria could not be applied due to limitations in data granularity [4]. Specifically, we were unable to exclude individuals with planned coronary, carotid or peripheral artery revascularisation, as well as those with NYHA Class IV heart failure. In addition, PAD was identified using diagnosis and procedure codes, rather than the symptomatic criteria used in the SELECT trial. HbA1c values were unavailable for approximately 60% of the SELECT‐like obesity cohort, meaning that some individuals with undiagnosed dysglycaemia may not have been identified through laboratory screening alone. However, diabetes was excluded using three complementary approaches, namely diagnosis codes, glucose‐lowering drug dispensations, and available HbA1c values, and two sensitivity analyses (a complete case analysis and a multiple imputation analysis) produced results consistent with the main analysis (Table S5), suggesting that missing HbA1c data did not materially influence our results. Furthermore, cohort entry required both survival and contact with healthcare services long enough for BMI to be documented after the qualifying cardiovascular event (mean time from qualifying event to inclusion: up to 7.3 years), introducing a form of survivorship conditioning. However, this design mirrors the SELECT trial, where participants were similarly enrolled years after their index cardiovascular event. As with all observational research, the accuracy of the data depends on the quality of recorded healthcare information, which may be incomplete or misclassified in some cases. However, the overall diagnostic validity of recorded hospital diagnoses in Sweden is generally high [23].

In conclusion, in a SELECT‐like cohort with obesity and established CVD, the risk of adverse cardiovascular outcomes was higher than in the SELECT trial, with an estimated NNT of 35 to prevent one MACE compared with 67 in the trial. Compared with the general population, patients with obesity and CVD experienced more than twice the risk of most cardiovascular outcomes, despite preventive pharmacotherapy. Those who would have been eligible for inclusion in the SELECT trial only comprised 3.4% of all individuals with obesity, and they experienced markedly higher rates of CVD than the broader obesity population. This indicates that whilst obesity contributes to elevated cardiovascular risk, the coexistence of obesity and established CVD identifies a particularly high‐risk group. These findings underscore the substantial disease burden and highlight the need for more effective preventive strategies, including the potential role of GLP‐1 receptor agonists in clinical practice.

## Author Contributions

All authors fulfil the ICMJE requirements for authorship. **V.R.:** contributed to the study conception and study design, participated in data interpretation, reviewed and approved the final manuscript. **H.N**. and **J.F.:** contributed to the study conception, study design and coordination, participated in data interpretation, reviewed and approved the final manuscript. **T.C.:** contributed to the study conception, study design and coordination, was responsible for data collection and data analysis, participated in data interpretation, helped to draft the manuscript, reviewed and approved the final manuscript. **M.K.S.:** contributed to the study conception, study design and coordination, was responsible for data collection, participated in data interpretation, reviewed and approved the final manuscript. **A.N.:** contributed to the study conception and study design, participated in data interpretation, reviewed and approved the final manuscript.

## Funding

This study was supported by Novo Nordisk Scandinavia AB, The Swedish Heart‐Lung Foundation (20251279), ALF Medicine, Region Stockholm (FoUI‐1002688) and Department of Research and Development, Region Kronoberg. Uppsala University supported through time allocation for M.K.S. in‐kind.

## Conflicts of Interest

V.R. had research support from the Department of Research and Development, Region Kronoberg for her time writing the manuscript. V.R. has received honoraria for expert group participation from Astra Zeneca, Novo Nordisk and Boehringer Ingelheim. M.K.S. has received honoraria for lectures from Amgen, AstraZeneca, Boehringer Ingelheim, CSL Vifor and Novo Nordisk, and contributed expertise to Boehringer Ingelheim, GSK, and Novo Nordisk advisory boards. A.N. has received personal honoraria for expert group participation, advisory board meeting, and lectures with presentations during the last 3 years from AstraZeneca, Novo Nordisk, Bayer and Boehringer Ingelheim. Previously from MSD Sweden and Eli Lilly. A.N. has received funding for research time from Capio S:t Görans hospital and from the Swedish Heart and Lung foundation. T.C. is an employee and shareholder at Sence Research AB, which is a company in biostatistics and epidemiology that received payment from Novo Nordisk for project management, data management and statistical analysis during the conduct of the study. J.F. and H.N. are employees of Novo Nordisk Scandinavia AB.

## Supporting information


**Table S1:** Overview of inclusion and exclusion criteria applied in the SELECT trial and the current study.
**Table S2:** Overview of definitions for comorbid conditions.
**Table S3:** Overview of definitions for treatments.
**Table S4:** Overview of definitions for outcomes.
**Table S5:** Sensitivity analyses for the impact of missing HbA1c data on MACE outcomes.

**Figure S1:** Flowchart of the derivation of the *SELECT‐like obesity cohort*, the General population cohort, and the General obesity cohort. BMI, body mass index; CVD, cardiovascular disease.

**Figure S2:** Number needed to treat (NNT) in the *SELECT‐like obesity cohort*, by BMI groups, based on the relative treatment effect observed in the SELECT trial. The presented NNTs are unweighted, in contrast to the weighted estimates reported in the main manuscript.

**Figure S3:** Number needed to treat (NNT) in the *SELECT‐like obesity cohort*, estimated from the relative treatment effect reported in the SELECT trial. NNT values are weighted to match the age and sex distribution of the SELECT trial population. MACE, major adverse cardiovascular event.

**Figure S4:** Time‐to‐First‐Event Analysis for outcomes. Cumulative incidence and hazard ratios weighted to harmonise with the age and sex distribution in the SELECT trial^1,3^ (competing risks taken into account). (A) Major Adverse Cardiovascular (CV) Event (competing risk: non‐CV death), (B) Cardiovascular death (competing risk: non‐CV death), (C) Myocardial infarction (competing risk: all‐cause death), (D) Stroke (competing risk: all‐cause death), (E) Heart failure composite (competing risk: all‐cause death), (F) Hospitalisation due to heart failure (competing risk: all‐cause death), and (G) all‐cause death (competing risk: all‐cause death). The definitions of all outcomes are provided in Methods and in Supporting Information.

## Data Availability

Relevant aggregated data and analytical code for this research project will be made available to Bona Fide Researchers upon reasonable request to M.K.S., following a legal assessment to ensure data confidentiality. Individual participant‐level data will not be shared. Requests can be made immediately after publication of this Article.
