# Supplementary material for: Cardiovascular Outcomes in a SELECT‐Like Obesity Cohort: Real‐World Insights From the Swedish AROS Database
Source: Diabetes Obes Metab. 2026 Jun 16;28(9):8057–71. doi: 10.1111/dom.70899 (PMC13448875; doi:10.1111/dom.70899)
Supplement: Supplementary file 1 [file DOM-28-8057-s001.pdf]

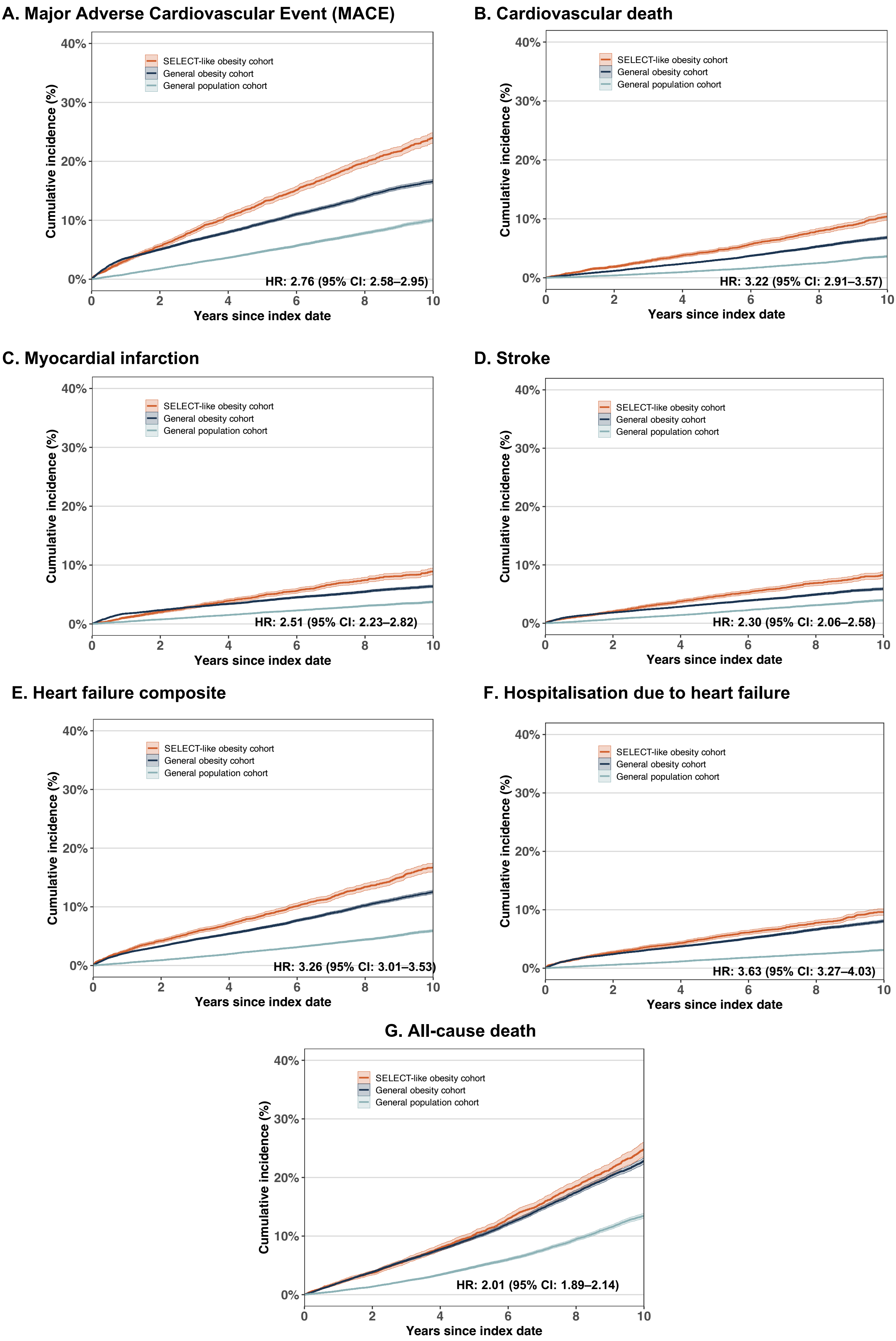
A. Major Adverse Cardiovascular Event (MACE)
SELECT-like obesity cohort
General obesity cohort
General population cohort
HR: 2.76 (95% CI: 2.58–2.95)
B. Cardiovascular death
HR: 3.22 (95% CI: 2.91–3.57)
C. Myocardial infarction
HR: 2.51 (95% CI: 2.23–2.82)
D. Stroke
HR: 2.30 (95% CI: 2.06–2.58)
E. Heart failure composite
HR: 3.26 (95% CI: 3.01–3.53)
F. Hospitalisation due to heart failure
HR: 3.63 (95% CI: 3.27–4.03)
G. All-cause death
HR: 2.01 (95% CI: 1.89–2.14)
Cumulative incidence (%)
Years since index date
0%
10%
20%
30%
40%
0
2
4
6
8
10
