# Supplementary material for: Cardiovascular Outcomes in a SELECT‐Like Obesity Cohort: Real‐World Insights From the Swedish AROS Database
Source: Diabetes Obes Metab. 2026 Jun 16;28(9):8057–71. doi: 10.1111/dom.70899 (PMC13448875; doi:10.1111/dom.70899)
Supplement: Supplementary file 2 [file DOM-28-8057-s002.pdf]

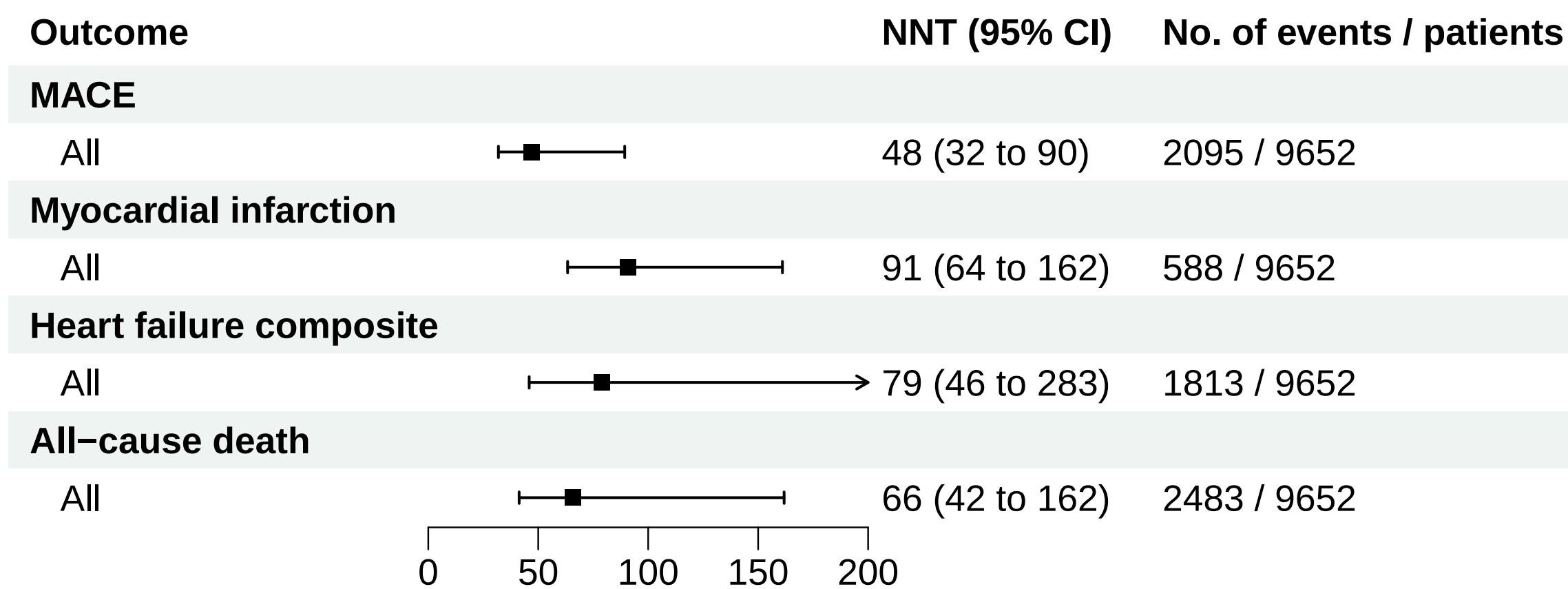
Outcome
NNT (95% CI)
No. of events / patients
MACE
All
48 (32 to 90)
2095 / 9652
Myocardial infarction
All
91 (64 to 162)
588 / 9652
Heart failure composite
All
79 (46 to 283)
1813 / 9652
All−cause death
All
66 (42 to 162)
2483 / 9652
0
50
100
150
200
