# Supplementary material for: Cardiovascular Outcomes in a SELECT‐Like Obesity Cohort: Real‐World Insights From the Swedish AROS Database
Source: Diabetes Obes Metab. 2026 Jun 16;28(9):8057–71. doi: 10.1111/dom.70899 (PMC13448875; doi:10.1111/dom.70899)
Supplement: Supplementary file 3 [file DOM-28-8057-s003.pdf]

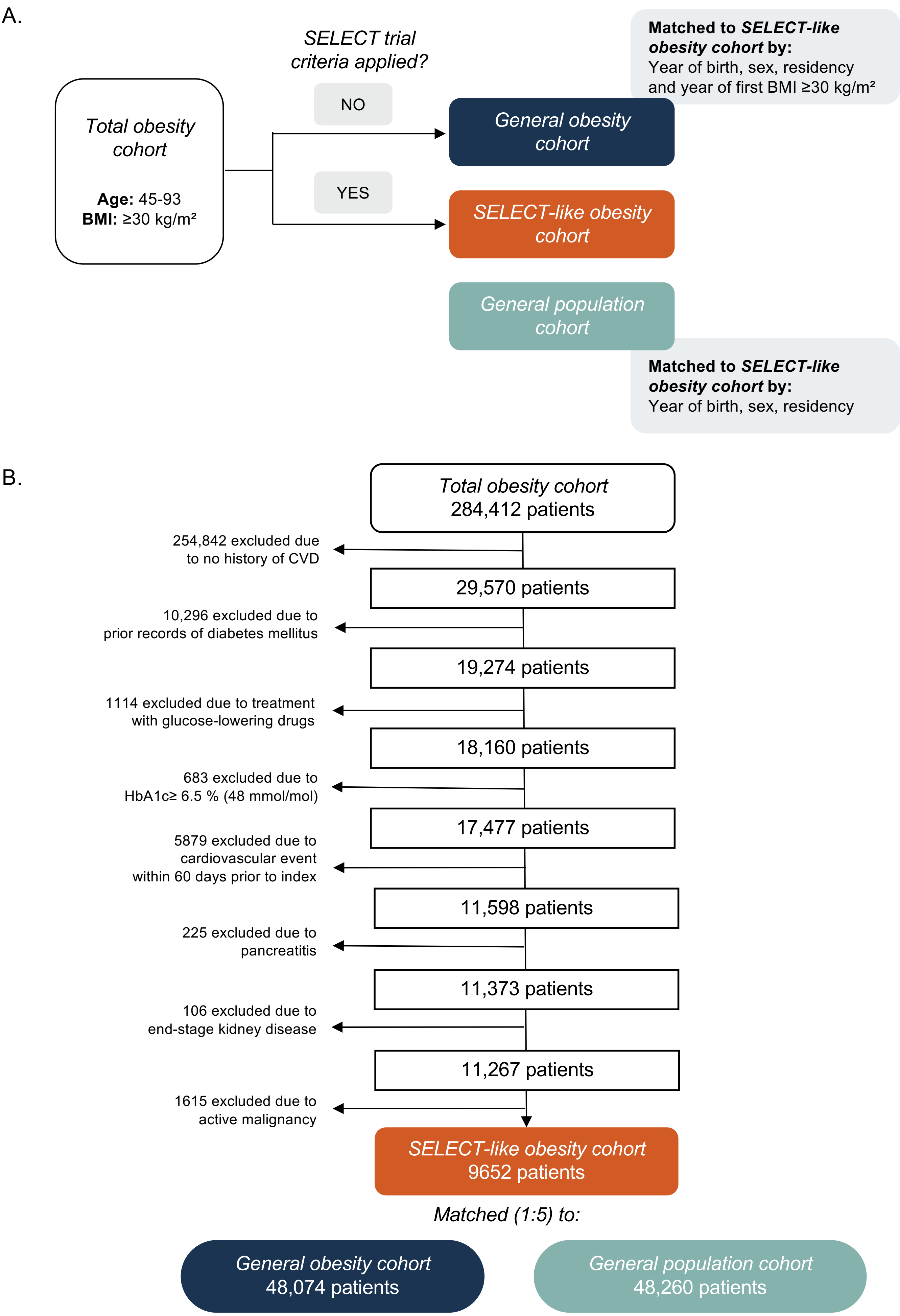
A.
SELECT trial criteria applied?
Matched to SELECT-like obesity cohort by:
Year of birth, sex, residency and year of first BMI ≥30 kg/m²
Total obesity cohort
Age: 45-93
BMI: ≥30 kg/m²
NO
YES
General obesity cohort
SELECT-like obesity cohort
General population cohort
Matched to SELECT-like obesity cohort by:
Year of birth, sex, residency
B.
Total obesity cohort 284,412 patients
254,842 excluded due to no history of CVD
29,570 patients
10,296 excluded due to prior records of diabetes mellitus
19,274 patients
1114 excluded due to treatment with glucose-lowering drugs
18,160 patients
683 excluded due to HbA1c≥ 6.5 % (48 mmol/mol)
17,477 patients
5879 excluded due to cardiovascular event within 60 days prior to index
11,598 patients
225 excluded due to pancreatitis
11,373 patients
106 excluded due to end-stage kidney disease
11,267 patients
1615 excluded due to active malignancy
SELECT-like obesity cohort 9652 patients
Matched (1:5) to:
General obesity cohort 48,074 patients
General population cohort 48,260 patients
