# Supplementary material for: Cardiovascular Outcomes in a SELECT‐Like Obesity Cohort: Real‐World Insights From the Swedish AROS Database
Source: Diabetes Obes Metab. 2026 Jun 16;28(9):8057–71. doi: 10.1111/dom.70899 (PMC13448875; doi:10.1111/dom.70899)

Outcome	NNT (95% CI)	No. of events / patients
**MACE**		
All	35 (24 to 66)	2095 / 9652
Class 1 obesity	34 (24 to 65)	1629 / 7645
Class 2 obesity	37 (25 to 71)	350 / 1546
Class 3 obesity	34 (22 to 70)	116 / 461
**Myocardial infarction**		
All	92 (64 to 164)	588 / 9652
Class 1 obesity	92 (64 to 165)	454 / 7645
Class 2 obesity	90 (60 to 178)	109 / 1546
Class 3 obesity	110 (66 to 332)	25 / 461
**Heart failure composite**		
All	45 (26 to 159)	1813 / 9652
Class 1 obesity	46 (27 to 162)	1379 / 7645
Class 2 obesity	42 (25 to 155)	332 / 1546
Class 3 obesity	41 (23 to 165)	102 / 461
**All−cause death**		
All	36 (23 to 88)	2483 / 9652
Class 1 obesity	36 (23 to 88)	1928 / 7645
Class 2 obesity	38 (24 to 95)	426 / 1546
Class 3 obesity	34 (21 to 89)	129 / 461

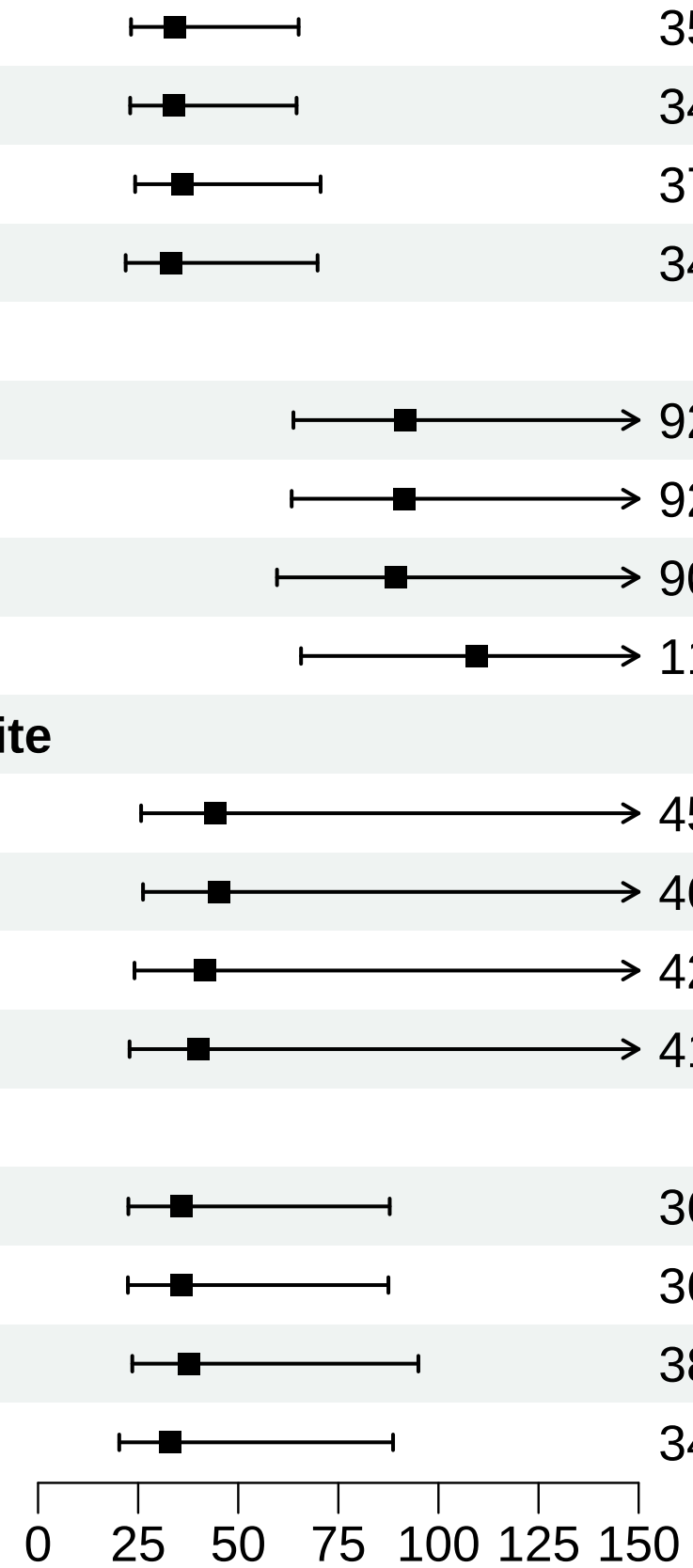

Supplement: Supplementary file 4 [file DOM-28-8057-s004.pdf]
