# Supplementary material for: Cardiovascular Outcomes in a SELECT‐Like Obesity Cohort: Real‐World Insights From the Swedish AROS Database
Source: Diabetes Obes Metab. 2026 Jun 16;28(9):8057–71. doi: 10.1111/dom.70899 (PMC13448875; doi:10.1111/dom.70899)
Supplement: Supplementary file 5 [file DOM-28-8057-s005.docx]

**Supplementary Material**

**Supplementary Tables**

**Supplementary Table 1.** Overview of inclusion and exclusion criteria applied in the SELECT trial and the current study.

|  | SELECT trial ^1^ | *SELECT-like obesity cohort* in AROS |
| --- | --- | --- |
| Inclusion criteria | | |
| Age | ≥45 | 45–93 |
| Sex | Male or female | |
| BMI | ≥27 kg/m^2^ | ≥30 kg/m^2^ |
| Cardiovascular-related | Prior MI ^a,b^ | |
|  | Prior stroke (ischemic or haemorrhagic stroke) ^a,b^ | |
|  | Symptomatic PAD, as evidenced by intermittent claudication with ankle–brachial index <0.85, peripheral arterial revascularisation procedure, or amputation due to atherosclerotic disease. | PAD^a,b^ |
| Exclusion criteria | | |
| Cardiovascular-related | MI within past 60 days prior to screening  Hospitalisation for unstable angina pectoris or transient ischemic attack within the past 60 days prior to the day of screening. | MI within past 60 days prior to index date. Hospitalisation for unstable angina pectoris^a^ or transient ischemic attack^a^ within the past 60 days prior to index date |
|  | Stroke within past 60 days prior to screening | Stroke^a^ within past 60 days prior to index date |
|  | Planned coronary, carotid, or peripheral artery revascularisation known on the day of screening. | Cannot be addressed using AROS data |
|  | Presently classified as being in New York Heart Association Class IV | Cannot be addressed using AROS data |
| Glycemia related | HbA_1c_ ≥48 mmol/mol (6.5%), as measured by the central laboratory at screening. | HbA_1c_ ≥48 mmol/mol (6.5%); data not available for all patients, see Table 1) |
|  | History of type 1 or type 2 diabetes (history of gestational diabetes is allowed) ^a^ | |
|  | Treatment with glucose-lowering agent(s) (including glucagon-like peptide-1 receptor agonists [GLP-1RAs] for indications other than type 2 diabetes mellitus) within 90 days before screening. | Treatment with anti-diabetic medication (ATC code A10) prior to index date. |
| General safety | History or presence of chronic pancreatitis. | History or presence of chronic pancreatitis^a^ |
|  | Presence of acute pancreatitis within the past 180 days prior to screening. | Presence of acute pancreatitis^a^ any time prior to index date |
|  | Personal or first-degree relative(s) history of multiple endocrine neoplasia type 2 or medullary thyroid carcinoma. | Cannot be addressed using AROS data |
|  | End stage kidney disease or chronic or intermittent haemodialysis or peritoneal dialysis. | End stage kidney disease^a^ recorded any time prior to index date |
|  | Presence or history of malignant neoplasms within the past 5 years prior to the day of screening. Basal and squamous cell skin cancer and any carcinoma in-situ are allowed. | Any record of an ICD-10 code within chapter C (malignant neoplasms) except for C44 prior to and including the index date. |
|  | Severe psychiatric disorder which in the investigator’s opinion could compromise compliance with the protocol. | Not applicable |
|  | Known or suspected hypersensitivity to trial product(s) or related products. | Cannot be addressed using AROS data |
|  | Previous participation in this trial. Participation is defined as randomisation. | Cannot be addressed using AROS data |
|  | Receipt of any investigational medicinal product within 30 days before screening. | Cannot be addressed using AROS data |
|  | Female who is pregnant, breastfeeding, or intends to become pregnant, or is of child- bearing potential and not using a highly effective contraceptive method. | Not addressed in AROS data |
|  | Any disorder, unwillingness, or inability, which in the investigator’s opinion, might jeopardise the patient’s safety or compliance with the protocol. | Not applicable |

Abbreviations: PAD, peripheral artery disease; MI, myocardial infarction.

a Identified using diagnosis and/or procedure codes listed in Supplementary table 2the AROS database.

b In the AROS database, diagnoses of MI, stroke or PAD had to be recorded in the primary position in inpatient care to be included as an inclusion criterion.

**Supplementary Table 2.** Overview of definitions for comorbid conditions.

| Condition | Diagnosis code (ICD-10) | Procedure code |
| --- | --- | --- |
| Coronary artery disease | I20-I25 | ·· |
| Myocardial infarction | I21 | ·· |
| Stroke | I60, I61, I63, I64 | ·· |
| Transient ischaemic attack | G45 | ·· |
| Peripheral artery disease | I70.0, I70.2 | PDE, PDF, PDH, PDN, PDQ, PDU, PDW, PEA, PEC, PEE, PEF, PEH, PEN, PEP, PEQ, PER, PET, PEU, PEW, PFA, PFB, PFE, PFH, PFN, PFP, PFQ, PFR, PFT, PFU, PFW, PGH, PGU, PGW, NFQ, NGQ, VH, VJ, VL |
| Chronic kidney disease (CKD) | N08.3, N17, N18, N19, E10.2, E11.2, E12.2, E13.2, E14.2, I12.0, I12.9, I13.1, I13.9, Z49.1, Z49.2, Z99.2, | ·· |
| Diabetes | E10, E11, E12, E13, E14 | ·· |
| Death due to cardiovascular cause | Death during study period with main cause of death diagnosis code starting with I | ·· |
| Hospitalisation due to heart failure | Inpatient with main diagnosis of I50 | ·· |
| Dyslipidaemia | E78 | ·· |
| Heart failure | I50 | ·· |
| Hypertension | I10, I11, I12, I13, I15 | ·· |
| Atrial fibrillation | I48 |  |
| Coronary revascularisation | ·· | FNA, FNB, FNC, FND, FNE, FNG00, FNG02, FNG05 |
| Angina pectoris | I20 | ·· |
| Chronic or acute pancreatitis | K85, K860, K861 | ·· |
| End-stage kidney disease | N18.5 + dialysis (Z49.1, Z49.2, Z99.2) + eGFR^*^ < 15 mL/min/1.73 m^2^ | ·· |
| Metabolic Dysfunction-Associated Steatotic Liver Disease (MASLD) | K760 |  |
| Cancer (excluding non-melanoma skin cancer) | C00-C97 (excluding C44) |  |
| Bariatric surgery | ·· | JDF |

** eGFR was available for the majority of patients (see Table 1) and was estimated using the revised Lund–Malmö equation*^2^*.*

**Supplementary Table 3.** Overview of definitions for treatments.

| Drug class | ATC codes |
| --- | --- |
| Beta blockers | C07 |
| ACE inhibitors | C09A, C09B |
| Angiotensin receptor blockers | C09C, C09D |
| Calcium channel blockers | C08C |
| Lipid lowering treatment | C10 |
| Statins | C10AA |
| Ezetimibe | C10AX09 |
| Fibrates | C10AB |
| PCSK-9 inhibitors | C10AX13, C10AX14 |
| Platelet aggregation inhibitors | B01AC |
| Acetylsalicylic acid | B01AC06 |
| P2Y12 inhibitors | B01AC04, B01AC22, B01AC24, B01AC25 |
| Other platelet aggregation inhibitors | B01AC07 |
| Anticoagulants | B01AA, B01AE07, B01AF01, B01AF02, B01AF03 |
| Vitamin K antagonists | B01AA |
| Direct oral anticoagulants | B01AE07, B01AF01, B01AF02, B01AF03 |
| Glucose-lowering drugs | A10 |
| Insulin | A10A |
| Non-insulin | A10B |
| Metformin | A10BA02, A10BD05, A10BD07, A10BD08, A10BD10, A10BD11, A10BD13, A10BD14, A10BD15, A10BD16, A10BD17, A10BD18, A10BD19, A10BD20 |
| Sulfonylureas | A10BB |
| DPP-4 inhibitors | A10BH, A10BD07, A10BD08, A10BD09, A10BD10, A10BD11, A10BD12, A10BD13 |
| GLP-1 agonists | A10BJ |
| SGLT2 inhibitors | A10BK, A10BD15, A10BD20 |
| Other non-insulin glucose-lowering drugs | A10BF, A10BX02, A10BG |
| Orlistat | A08AB01 |
| Bupropion/naltrexon | A08AA62 |
| Liraglutide | A10BJ02 (plus filtering on Saxenda product name) |

**Supplementary Table 4.** Overview of definitions for outcomes

| Outcome | Definition |
| --- | --- |
| MACE | MACE was defined as a composite of cardiovascular death, myocardial infarction ^a^, or stroke ^a^. |
| Cardiovascular death | Death during study period with main cause of death diagnosis code starting with ICD-10 code: I (diseases of the circulatory system) |
| Myocardial infarction | ICD-10: I21^a^ |
| Stroke | ICD-10: I60, I61, I63, I64^a^ |
| Heart failure composite | Hospitalisation for heart failure or cardiovascular death^b^ |
| Hospitalisation due to heart failure | Hospitalisation with main diagnosis of ICD10: I50 |
| All-cause death | Death due to any cause |

*^a^ required hospitalisation with the condition recorded as the primary discharge diagnosis.*

*^b^ A validity of 95% has previously been described for the diagnosis of heart failure in the first position in the hospital discharge register.* ^3^

**Supplementary Table 5.** Sensitivity analyses for the impact of missing HbA1c data on MACE outcomes.

In the main analysis, patients with an HbA1c ≥48 mmol/mol prior to the index date were excluded, but 59.4% of the cohort had no HbA1c measurement available within three years of the index date and could therefore not be screened biochemically for diabetes. To assess the potential impact of undiagnosed diabetes in this group, we conducted two complementary sensitivity analyses.

In the complete case analysis, the cohort was restricted to the 3,916 patients (40.6%) with a confirmed HbA1c <48 mmol/mol within three years prior to the index date, and the 4-year MACE cumulative incidence and NNT were re-estimated in this subgroup. In the multiple imputation analysis, missing HbA1c values were imputed using predictive mean matching (10 imputations, 10 iterations) based on age, sex, BMI, region, eGFR, cardiovascular disease status, diabetes diagnosis, glucose-lowering drug use, and prevalent comorbidities (hypertension, heart failure, myocardial infarction, stroke, peripheral artery disease). Imputation was performed at an early stage of cohort assembly, prior to applying the cardiovascular disease requirement and diabetes-related exclusions. In each imputed dataset, all inclusion and exclusion criteria were then applied sequentially, including exclusion of individuals with imputed HbA1c ≥48 mmol/mol. On average, 1,804 patients per imputation were excluded due to observed or imputed HbA1c ≥48, yielding a mean final cohort size of 9,313 (range 9,272–9,358). The 4-year MACE cumulative incidence was estimated using the Aalen-Johansen estimator with non-cardiovascular death as a competing risk, and results were pooled using Rubin's rules. The fraction of missing information was 8.5%. The NNT was derived by applying the SELECT trial hazard ratio (HR 0.80, 95% CI 0.72–0.90) to the pooled baseline risk. Both sensitivity analyses produced results consistent with the main analysis, suggesting that the potential presence of undiagnosed diabetes among individuals with missing HbA1c values did not materially influence the findings.

| **Analysis** | **N** | **4-year MACE**  **cumulative incidence, % (95% CI)** | **NNT (95% CI)** |
| --- | --- | --- | --- |
| Main analysis | 9,652 | 14.60 (13.86–15.35) | 35 (24–66) |
| Complete case | 3,916 (40.6%) | 13.25 (12.10–14.40) | 38 (26–73) |
| Multiple imputation | 9,313 (mean; range 9,272–9,358) | 14.64 (13.82–15.46) | 35 (24–65) |

**Supplementary Figures**

**Supplementary Figure 1.** Flowchart of the derivation of the *SELECT-like obesity cohort,* the *General population cohort*, and the *General obesity cohort*. BMI, body mass index; CVD, cardiovascular disease.

**Supplementary Figure 2.** Number needed to treat (NNT) in the *SELECT-like obesity cohort*, by BMI groups, based on the relative treatment effect observed in the SELECT trial. The presented NNTs are unweighted, in contrast to the weighted estimates reported in the main manuscript.

**Supplementary Figure 3.** Number needed to treat (NNT) in the *SELECT-like obesity cohort*, estimated from the relative treatment effect reported in the SELECT trial. NNT values are weighted to match the age and sex distribution of the SELECT trial population. MACE, major adverse cardiovascular events.

**Supplementary Figure 4.** Time-to-First-Event Analysis for outcomes. Cumulative incidence and hazard ratios weighted to harmonise with the age and sex distribution in the SELECT trial ^1,4^ (competing risks taken into account). (A) Major Adverse Cardiovascular (CV) Event (competing risk: non-CV death), (B) Cardiovascular death (competing risk: non-CV death), (C) Myocardial infarction (competing risk: all-cause death), (D) Stroke (competing risk: all-cause death), (E) Heart failure composite (competing risk: all-cause death), (F) Hospitalisation due to heart failure (competing risk: all-cause death), and (G) all-cause death (no competing risk). The definitions of all outcomes are provided in Methods and in Supplementary Material.

**References**

1.	Lingvay I, Brown‐Frandsen K, Colhoun HM, et al. Semaglutide for cardiovascular event reduction in people with overweight or obesity: SELECT study baseline characteristics. *Obesity*. 2023;31(1):111-122. doi:10.1002/oby.23621

2.	Björk J, Grubb A, Sterner G, Nyman U. Revised equations for estimating glomerular filtration rate based on the Lund-Malmö Study cohort. *Scand J Clin Lab Invest*. 2011;71(3):232-239. doi:10.3109/00365513.2011.557086

3.	Ingelsson E, Ärnlöv J, Sundström J, Lind L. The Validity of a Diagnosis of Heart Failure in a Hospital Discharge Register. *Eur J Heart Fail*. 2005;7(5):787-791. doi:10.1016/j.ejheart.2004.12.007

4.	Lincoff AM, Brown-Frandsen K, Colhoun HM, et al. Semaglutide and Cardiovascular Outcomes in Obesity without Diabetes. *N Engl J Med*. 2023;389(24):2221-2232. doi:10.1056/NEJMoa2307563
